# Time-Frequency Analysis of Non-Stationary Biological Signals with Sparse Linear Regression Based Fourier Linear Combiner

**DOI:** 10.3390/s17061386

**Published:** 2017-06-14

**Authors:** Yubo Wang, Kalyana C. Veluvolu

**Affiliations:** 1 School of Life Science and Technology, Xidian University, Xi’an 710071, ShanXi, China; ybwang@xidian.edu.cn; 2 School of Electronics Engineering, Kungpook National University, Daegu 702-701, Korea

**Keywords:** time-frequency decomposition, truncated fourier series model, sparse linear regression, *ℓ*_1_ regularization, ADMM

## Abstract

It is often difficult to analyze biological signals because of their nonlinear and non-stationary characteristics. This necessitates the usage of time-frequency decomposition methods for analyzing the subtle changes in these signals that are often connected to an underlying phenomena. This paper presents a new approach to analyze the time-varying characteristics of such signals by employing a simple truncated Fourier series model, namely the band-limited multiple Fourier linear combiner (BMFLC). In contrast to the earlier designs, we first identified the sparsity imposed on the signal model in order to reformulate the model to a sparse linear regression model. The coefficients of the proposed model are then estimated by a convex optimization algorithm. The performance of the proposed method was analyzed with benchmark test signals. An energy ratio metric is employed to quantify the spectral performance and results show that the proposed method Sparse-BMFLC has high mean energy (0.9976) ratio and outperforms existing methods such as short-time Fourier transfrom (STFT), continuous Wavelet transform (CWT) and BMFLC Kalman Smoother. Furthermore, the proposed method provides an overall 6.22% in reconstruction error.

## 1. Introduction

The recent advances in technology have paved the way for deployment of reliable biological sensors in clinical practice. A wide variety of such sensors have been developed to measure biosignals that reflect various underlying physiological phenomena. For example, gyroscope and accelerometers are employed for pathological and physiological tremor signal measurement [1], accelerometers are employed for cardiac mechanical vibrations monitoring [2], infrared sensors are employed for respiration motion monitoring [3], and common electrodes are employed for brain and heart electrical activity measurement [4,5]. In order to adequately interpret the signals and make useful observations, a proper understanding of the involved phenomena and their influence on the signals is necessary. However, most of the physiological signals are non-stationary due to the complex nature of the biological systems. Very subtle changes in the time-frequency characteristics of these signals can potentially correspond to an underlying condition. Therefore, analysis of biosignals require high resolution time-frequency decomposition methods to effectively detect these subtle changes.

The frequency domain information of a signal is usually obtained by applying the well-known Fourier transform. However, its strong assumption on the stationarity of the signal is often violated by the signals that are collected from biological or biomedical systems. To handle the non-stationarity of such signals, time-frequency decomposition methods are usually employed for obtaining time-frequency mapping of such signals [4,5,6,7]. The time-varying characteristics are thus analyzed in both time and frequency domains. Commonly employed time-frequency decomposition methods can be categorized into parametric and non-parametric depending on whether a signal model is required [8]. Fourier transform and Wavelet transform based methods are the typical parametric time-frequency decomposition methods, whereas the autoregressive model is the most popular choice for parametric time-frequency decomposition [5,6]. For non-uniformly sampled data, the windowed Lomb periodogram is usually employed [9,10].

If the signal is uniformly sampled, and its characteristics change slowly over time, we can safely assume that the stationarity holds for a short time interval. The Fourier transform is then applied to the portion of the signal, which is extracted from the original signal by multiplying it with a properly selected window function. This procedure is known as STFT [11]. Although the global stationary requirement of the signal is relaxed by assuming piecewise stationarity, the optimal window length is often difficult to be determined.

In STFT, the temporal and spectral resolutions are proportional to each other. The product of spectral resolution and temporal resolution is bounded by a fixed value [12]. If the window length is fixed, a trade-off between temporal and spectral resolutions must be made. The imbalance between spectral and temporal resolutions may cause a leakage effect in the obtained spectrogram [11,12]. Moreover, the window function needs to be tailored according to the frequency range of interest to achieve the desired temporal and spectral resolution. The CWT solves this problem by decomposing the signal into a basis of dilated and shifted version of a pre-defined mother wavelet [13]. By shrinking the mother wavelet, CWT attains a better trade-off in temporal and spectral resolution as compared to STFT.

Apart from the non-parametric time-frequency decomposition methods such as STFT and CWT, the time-frequency mapping of a signal can also be obtained by fitting a parametric model to the signal and then transforming the estimated coefficients of the model into its corresponding time-frequency plane. As shown in [14], the time-frequency mapping can be obtained from the estimated coefficients of the autoregressive (AR) model. To account for the time-varying characteristics of the signal, the AR coefficients are estimated through adaptive algorithms such as least mean square (LMS) and recursive least mean square (RLS) [15]. With the assumption that the AR process is driven by the Gaussian noise, the state-space form of the AR model can be formulated with the Kalman filter as the optimal estimator [16]. The Kalman smoother is also employed for achieving better results in offline analysis [15,17]. To further refine the estimation of the AR model, the expectation maximization algorithm is employed for identification of the state transition matrix [17] and particle filter to account for the non-Gaussian noise case [18]. The temporal resolution of AR based time-frequency decomposition is guaranteed by the employed adaptive algorithm, whereas the spectral resolution is strongly affected by the AR model order. As the commonly applied order selection methods rely on the estimation error, the selected order is only optimal for either reconstruction or prediction and does not offer better time-frequency mapping [14].

To mitigate the sensitivity of spectral resolution on model order, the frequency characteristics of the signal need to be considered in the signal model. One such model is BMFLC [19,20]. The BMFLC divides the frequency band of interest with a fixed frequency gap and adopts a truncated Fourier series as the signal model. The estimated Fourier coefficients for each frequency component can thus be used to form the time-frequency mapping of the signal. It was shown in [21] that the BMFLC with adaptive filter algorithms, such as least-mean square and Kalman filter, was successful for time-frequency decomposition of motion induced Electroencephalography (EEG) signal in real time. The estimated time-frequency mapping can be fine-tuned with the help of a smoother procedure. The comparison study in [21] also suggests that the BMFLC with Kalman smoother (BMFLC-KS) provides better temporal and spectral resolutions than STFT.

In the scenario where two frequency components lie closely to each other in spectral domain [22], BMFLC requires a frequency gap that is at least equal to the distance between the two frequency components to differentiate them [21]. However, the dimension of states in BMFLC increases with the increased spectral resolution (i.e., smaller frequency gap), which further increases computational complexity. Moreover, the high-dimensionality also causes the adaptive filter paired with BMFLC to fail at providing an accurate time-frequency decomposition [21]. In this work, we address this problem by incorporating sparse linear regression with BMFLC.

Recently the sparse linear regression model has found numerous applications in signal processing [23,24,25]. The sparse linear regression uses $\ell_{1}$ norm to regularize the regression coefficients. It has been proven to generate a better signal model for non-stationary signals [26]. In [27], the sparse linear regression model is used for estimating the frequency-hopping signal in communication applications. In [28], the AR coefficients are expanded onto a redundant set of basis functions, which simplifies the identification of time-varying AR coefficients into time-invariant case. Then, a sparse-aware regression method [29] is employed to find the most informative one in the redundant model in order to improve the overall estimation performance. Its application to phase retrieval of sparse signal is shown in [30].

The large dimension of states in BMFLC caused by increased spectral resolution necessitates the imposition of sparsity on the model of BMFLC under the assumption that only a few coefficients change at any time instant. As BMFLC inherits linearity from the Fourier series, the BMFLC model can thus be modeled in the form of sparse linear regression model. Similar to [22], the super-resolution can be achieved by estimating the amplitude from a redundant set of frequencies.

In this work, we model the BMFLC in the form of a sparse linear model and impose $\ell_{1}$ constraint on the model coefficients. The convex optimization algorithm is then employed to estimate model coefficients. The estimation accuracy of the model is compared with BMFLC Kalman smoother (BMFLC-KS). The time-frequency decomposition performance of the proposed model is compared with STFT, CWT and BMFLC-KS on four synthetic signals. An energy ratio metric is also employed to demonstrate the effectiveness of the proposed model.

## 2. Methodology

In this section, we first review the existing BMFLC signal model. After the identification of the sparsity in the model, the formulation of the proposed sparse-BMFLC is discussed in this section. The model coefficients are then estimated with the alternating direction methods of the multiplier (ADMM) method.

### 2.1. Model of Sparse-BMFLC

The band-limited multiple Fourier linear combiner (BMFLC) divides the frequency band of interest, [$f_{1},f_{M}$], into *M* divisions with a fixed frequency gap Δf and estimates the signal amplitude according to the formation of the Fourier series [4,21]. The signal model can be described as

$$\begin{array}{c} y_{n}=\sum_{i=1}^{M}a_{n,i}\sin\left(2\pi f_{i}n\right)+b_{n,i}\cos\left(2\pi f_{i}n\right),\;i=1,2,\cdots ,M, \end{array}$$

where $y_{n}$ is the signal at time instant *n*, where n∈[1,2,⋯,N]. *N* is the total number of samples of the signal to be analyzed. $a_{n,i}$ and $b_{n,i}$ are the Fourier coefficients corresponding to the frequency $f_{i}$. Note that, unlike the traditional Fourier series, which is defined in the complex domain, the BMFLC uses trigonometric functions and therefore is only defined in real space $R^{d}$. The time-frequency mapping of BMFLC is obtained by estimating the coefficients at each time instant via Kalman filter or Kalman smoother [21].

The dimension *d* of the BMFLC is defined as the number of coefficients to be estimated and is given as $2(f_{1}-f_{M})/\Delta f=2M$. As we increase the spectral resolution, which is equivalent to reduction in the frequency gap Δf, *d* increases with the rate of 1/Δf. In [21], the optimal frequency gap is selected based on the application requirements. For example, the motion induced EEG signal requires amplitude estimation in the frequency range of 7 Hz to 14 Hz, which results in a 28 dimension state for BMFLC. If Δf is chosen to be 0.1 Hz, then the dimension increases to 140. Despite the high estimation accuracy, the obtained time-frequency mapping was not accurate [21].

If we assume that there are *N* samples of data, the BMFLC model can be re-formulated as follows. First, we denote $w_{n}=\left[\sin\left(2\pi f_{1}n\right),\cdots ,\sin\left(2\pi f_{M}n\right),\cos\left(2\pi f_{1}n\right),\cdots ,\cos\left(2\pi f_{M}n\right)\right]$, $x_{n}=\left[a_{n,1},\cdots ,a_{n,M},b_{n,1},\cdots ,b_{n,M}\right]$ and $Y=[y_{1},y_{2},\cdots ,y_{N}]$. Then, W and X are given as:

(1) $$W=\begin{array}{c} \;\overset{\;(N-1)d\;}{︷}\\ \left[\begin{array}{ccccc} w_{1} & 0 & 0 & \cdots & 0\\ 0 & w_{2} & 0 & \cdots & 0\\ \vdots & & \cdots & & \vdots \\ 0 & 0 & 0 & \cdots & w_{N} \end{array}\right], \end{array}$$

(2) $$\begin{array}{c} X={\left[x_{1},x_{2},\cdots ,x_{N}\right]}^{T}, \end{array}$$

where the superscript ${}^{T}$ denotes the matrix transpose. By construction, X is the time-frequency mapping of the signal. The BMFLC in Equation (1) can be re-written in the linear regression form as:

(3) $$\begin{array}{c} Y=WX+V, \end{array}$$

where V follows a multidimensional Gaussian distribution N(0,Σ). There are two types of sparsity that can be identified in Equation (3). With a very small frequency gap Δf, only a few frequency components are non-zero at any time instant. Therefore, the vector X is sparse. Furthermore, it is unlikely that the majority of states varies dynamically over time and more likely that sparsity also exists during the transition between two consecutive instances—i.e., $x_{n,i}-x_{n-1,i}$ is also sparse. Thus, Equation (3) forms a sparse linear regression model.

The solution of Equation (3) depends on the structure of matrix W. As given in Equation (1), the matrix W has the dimension of N×Nd. If more frequency components are required, *d* can be very large, which leads to Nd≫N. Therefore, a unique solution for X does not exist. The sparse linear model is ill-posed. Nonetheless, it is always possible to find a solution that satisfies the previously identified sparsity constraints. The sparsity in a vector is usually measured by the $\ell_{0}$ norm. Thus, the solution for Equation (3) can be found by solving the following optimization problem:

(4) $$\begin{array}{c} X^{*}=\underset{X}{\arg\min}\left[\begin{array}{c} \frac{1}{2}{∥Y-WX∥}_{2}^{2}+\lambda_{1}{∥X∥}_{0}+\lambda_{2}{∥CX∥}_{0} \end{array}\right], \end{array}$$

where C can be defined similar to [26] as $C={\left[c_{0},c_{1},\cdots ,c_{\left(N-1)(d-1\right)}\right]}^{T}$, $c_{0}=\left[-1,\overset{d-1}{\overset{︷}{0,\cdots ,0}},1,\overset{\left(N-1)(d-1\right)}{\overset{︷}{0,\cdots ,0}}\right]$, where the subscript of $c_{m}$ indicates right shifting $c_{0}$ by m∈[1,(N−1)(d−1)] positions. Therefore, the sparsity in $x_{n,i}-x_{n-1,i}$ now lies in the matrix product CX. Furthermore, ${∥\cdot ∥}_{2}$ and ${∥\cdot ∥}_{0}$ denote the $\ell_{2}$ and $\ell_{0}$ norm of a vector, respectively. It is clear that the first term on the right-hand side of the equation is the least square error of the regression model. The second and the third terms are used to regularize the solution towards sparsity [31]. As the $\ell_{0}$ norm of a vector is not differentiable, it is difficult to apply any optimization algorithm to find the solution of such models. The $\ell_{1}$ norm is instead used for algorithmic purpose [26]. The $\ell_{1}$ regularized Equation (4) also named as the fused *lasso* regression [32] is given by:

(5) $$\begin{array}{c} X^{*}=\underset{X}{\arg\min}\left[\begin{array}{c} \frac{1}{2}{∥Y-WX∥}_{2}^{2}+\lambda_{1}{∥X∥}_{1}+\lambda_{2}{∥CX∥}_{1} \end{array}\right], \end{array}$$

where the positive scalars $\lambda_{1}$ and $\lambda_{2}$ in Equation (5) are employed to balance the sparsity between X and CX, respectively. As shown in [26], the values of $\lambda_{1}$ and $\lambda_{2}$ affect the estimation significantly. The general guidance for tuning these two parameters can be found in [27].

### 2.2. ADMM Solution for Sparse-BMFLC

Equation (5) is a convex optimization on X, which can be solved by various algorithms. However, the computational load increases as the dimension of W grows. It is shown in [33] that the alternating direction methods of multiplier (ADMM) algorithm can be easily extended to a distribution computation scheme. Thus, it makes the algorithm well suited to application scenarios where computational speed is the major concern.

We follow the procedure in [27] for ADMM to solve the fused *lasso* problem in Equation (5). We first define the auxiliary variables z and u. Then, Equation (5) can be transformed into a constraint optimization problem given as follows:

(6) $$\begin{array}{c} \begin{array}{c} \left[X^{*},z^{*},u^{*}\right]=\underset{X,z,u}{\arg\min}\left[\begin{array}{c} \frac{1}{2}{∥Y-WX∥}_{2}^{2}+\lambda_{1}{∥z∥}_{1}+\lambda_{2}{∥u∥}_{1} \end{array}\right],\\ \mathrm{subject}\;\mathrm{to}\;z=X,u=CX. \end{array} \end{array}$$

The Lagrange function is employed to account for the constraints in Equation (6). Assigning Lagrange multiplier (ϵ,μ) for the equality constraints, we can obtain the following Lagrange equation:

(7) $$\begin{array}{cc} L(X,z,u,\epsilon ,\mu )= & \frac{1}{2}{∥Y-WX∥}_{2}^{2}+\lambda_{1}{∥z∥}_{1}+\lambda_{2}{∥u∥}_{1}+\epsilon \left(X-z\right)\\ & +\mu \left(CX-u\right)+\frac{q}{2}{(∥X-z∥}_{2}^{2}{+(∥CX-u∥}_{2}^{2}). \end{array}$$

The ADMM finds the solution for Equation (7) by first assigning an initial condition for $z^{0},u^{0},\epsilon^{0},\mu^{0}$ and picks any positive number for *q*, and then solves $X^{i}$ recursively as:

(8) $$X^{i}=\underset{X}{\arg\min}\;L\left(X,z^{i-1},u^{i-1},\epsilon^{i-1},\mu^{i-1}\right).$$

Using the estimated $X^{i}$, the updated rule for $z^{i},u^{i}$ is obtained similarly. Finally, the ADMM identifies the solution for Equation (7) by using the following set of recursive equations:

(9) $$\begin{array}{ccc} X^{i} & = & {\left(W^{T}W+qC^{T}C+qI_{Nd}\right)}^{-1}\\ & & (W^{T}Y-\epsilon^{i-1}-C^{T}\mu^{i-1}+qz^{i-1}+qC^{T}u^{i-1}), \end{array}$$

(10) $$\begin{array}{ccc} z^{i} & = & \mathrm{shrink}(X^{i}+\frac{\epsilon^{i-1}}{q},\frac{\lambda_{1}}{q}), \end{array}$$

(11) $$\begin{array}{ccc} u^{i} & = & \mathrm{shrink}(CX^{i}+\frac{\mu^{i-1}}{q},\frac{\lambda_{2}}{q}), \end{array}$$

(12) $$\begin{array}{ccc} \epsilon^{i} & = & \epsilon^{i-1}+q\left(X^{i}-z^{i}\right), \end{array}$$

(13) $$\begin{array}{ccc} \mu^{i} & = & \mu^{i-1}+q\left(CX^{i}-u^{i}\right), \end{array}$$

where $I_{Nd}$ denotes an identity matrix with dimension of Nd. The shrinkage operator employed in Equations (10) and (11) is defined as:

$$\begin{array}{ccc} e_{k}^{i} & = & \mathrm{shrink}(x_{k}^{i}+\frac{\eta_{k}^{i-1}}{q},\frac{\lambda }{q})\\ & = & \left\{\begin{array}{c} 0,\;\mathrm{if}\;x_{k}^{i}+\frac{\eta_{k}^{i-1}}{q}=0,\\ \frac{x_{k}^{i}+q^{-1}\eta_{k}^{i-1}}{|x_{k}^{i}+q^{-1}\eta_{k}^{i-1}|}\max(|x_{k}^{i}+\frac{\eta_{k}^{i-1}}{q}\left|-\frac{\lambda }{q},0\right),\;\mathrm{otherwise}, \end{array}\right. \end{array}$$

where |a| is the absolute value, and $e_{k}^{i}$ denotes the *k*th dimension of the vector e in *i*th iteration. Note that the shrinkage operator is applicable to all dimensions of the vector. Also notice that, as the matrix inversion in Equation (9) does not change over iterations, we can pre-calculate and store its value to reduce the computational complexity of the ADMM algorithm. For detailed derivation of ADMM, please refer to [27,33,34].

### 2.3. Time-Frequency Decomposition from Sparse-BMFLC

As the model of BMFLC breaks the Fourier coefficients of a frequency component into its corresponding sine and cosine parts and estimates their amplitudes separately, the following formulation is employed for time-frequency decomposition of the signal:

(14) $$\begin{array}{c} P\left(t,f\right)=\left[\begin{array}{ccc} \frac{\sqrt{a_{1,1}^{*2}+b_{1,1}^{*2}}}{2} & \cdots & \frac{\sqrt{a_{n,1}^{*2}+b_{n,1}^{*2}}}{2}\\ \vdots & & \vdots \\ \frac{\sqrt{a_{1,M}^{*2}+b_{1,M}^{*2}}}{2} & \cdots & \frac{\sqrt{a_{n,M}^{*2}+b_{n,M}^{*2}}}{2} \end{array}\right], \end{array}$$

where $a_{n,i}^{*}$ and $b_{n,i}^{*}$ denote the amplitude estimation of the frequency $f_{i}$ at time instant *n*. Its value is extracted from the solution $X^{*}$.

## 3. Results

In this section, the performance of the developed sparse-BMFLC is evaluated with benchmark test signals. To analyze and quantify the performance of the time-frequency decomposition of the proposed sparse-BMFLC, it is compared to well known methods such as STFT and CWT.

### 3.1. Synthetic Signal

We select the following standard test signals to test the spectral and temporal resolutions:

$$\begin{array}{c} S_{1}\left(t\right)=\left\{\begin{array}{c} {2|t|}^{0.5}\sin\left(2\pi 8t\right)+4{\left|t\right|}^{0.5}\sin\left(2\pi 10t\right);\;0<t\leq 3,\\ {4|t|}^{0.25}\sin\left(2\pi 6t\right)+2{\left|t\right|}^{0.25}\sin\left(2\pi 12t\right);\;3<t\leq 6, \end{array}\right.\\ S_{2}\left(t\right)=\left\{\begin{array}{c} 2\sin\left(2\pi 8t\right)+4\sin\left(2\pi 10t\right);\begin{array}{c} 0<t\leq 2,\\ \;4<t\leq 6, \end{array}\\ 0,\;\;\;\;\;\;\;\;\;\;\;\;\;\mathrm{otherwise}, \end{array}\right.\\ S_{3}\left(t\right)=\sin\left(2\pi tf\left(t\right)\right);\;0<t\leq 6, \end{array}$$

where f(t)=5/3t+5 indicates a linear frequency sweep that starts from 5 Hz at t=0 and ends at 10 Hz at t=6 s. The sudden change that occurs at 3 s in $S_{1}\left(t\right)$ tests the frequency tracking performance. The amplitude modulation together with the two closer frequency components tests both the temporal and spectral resolutions. The amplitude of the signal $S_{2}\left(t\right)$ is set to zero in the middle section of the signal. The sudden change in amplitude poses a challenge, especially for the adaptive filter algorithms to adapt to true time-frequency mapping of the signal. As the BMFLC requires a proper setting of Δf to obtain optimal spectral resolution, the following chirp signal is employed to study the effect of frequency mismatch in the signal. To highlight the performance compared to the best existing BMFLC based method, BMFLC-KS [21] is also included for comparison in this section.

### 3.2. Parameter Selection

For the study, the parameters chosen for all methods are: $f_{1}=1$ Hz, $f_{M}=15$ Hz and Δf=0.5 Hz. The parameters $\lambda_{1}$ and $\lambda_{2}$ in sparse-BMFLC are difficult to select and tune. Based on the parameter selection guidelines provided in [27], the maximum theoretical value for both parameters are estimated. Then, $\lambda_{1}=0.01\lambda_{1}^{max}$ and $\lambda_{2}=0.05\lambda_{2}^{max}$ are selected empirically for their optimal performance. For BMFLC-KS, the diagonal elements of the state transition covariance matrix are all chosen to be 0.001 and the variance of measurement is set to 0.01. In STFT, the Gaussian window function is employed to obtain the optimal temporal and spectral resolutions. The window length is set to match the desired frequency resolution. For CWT, the Morlet mother wavelet with center frequency of 1.5 Hz is selected.

### 3.3. Estimation Performance of Sparse-BMFLC

**Figure 1:**
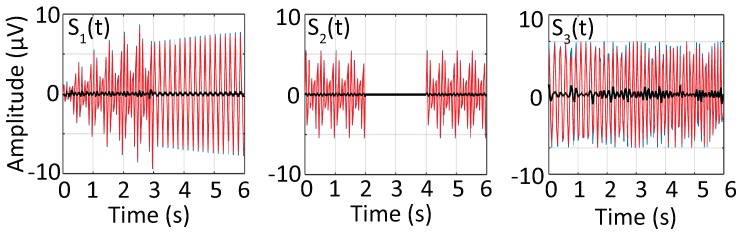
Estimation performance of sparse-BMFLC for all three synthesized signal $S_{1}\left(t\right)$, $S_{2}\left(t\right)$ and $S_{3}\left(t\right)$. The true signal together with the estimated signal and corresponding error are shown.

After obtaining the optimal solution $X^{*}$, one can obtain the amplitude estimate of the signal according to Equation (3). The original signals together with their corresponding reconstruction errors are shown in Figure 1. Results show that the proposed method can accurately estimate the modulated amplitude in $S_{1}\left(t\right)$ and sudden amplitude changes in $S_{2}\left(t\right)$ as shown in Figure 1. In the presence of frequency mismatch, the performance slightly decreases.

The overall estimation performance of sparse-BMFLC is quantified with percentage RMS error, that is defined as:

(15) $$\begin{array}{c} RMS\%=\frac{RMS(Y-\hat{Y})}{RMS(Y)}\times 100, \end{array}$$

where $RMS\left(Y\right)=\sqrt{\sum_{i=1}^{N}y_{i}^{2}/N}$, *i* indicates the number of samples and $\hat{Y}$ is the estimated signal. We also included the results of BMFLC-KS for comparison purposes. The results are tabulated in Table 1. The mean reconstruction error is 2.71% and 6.22% for BMFLC-KS and Sparse-BMFLC, respectively. Both methods can reconstruct the original signal with less than 10% error, which confirms the ability of the BMFLC based model in modeling the signal. Similar to Figure 1, the error for the sparse-BMFLC depends on the signals. We noticed that the performance of the sparse-BMFLC depends on the complexity of the signal. As shown in the second row of Table 1, the lowest estimation error was obtained in $S_{1}\left(t\right)$, which contained two frequency components, whereas the highest error occurred when applied to the chirp signal $S_{3}\left(t\right)$. It can be noticed that the Sparse-BMFLC has a large reconstruction error for all three synthetic signals. This is due to the fact that the optimal solution of Sparse-BMFLC is obtained when both $\ell_{1}$ and $\ell_{2}$ norms are minimized. Hence, the obtained solution differs from the one obtained when only $\ell_{2}$ norm of reconstruction error is minimized. Therefore, it is expected for Sparse-BMFLC to have a larger reconstruction error as compared to BMFLC-KS, which only minimizes the $\ell_{2}$ norm. The effect of estimation accuracy on the time-frequency decomposition will be discussed in the following section.

### 3.4. Time-Frequency Decomposition of the Synthesized Signal

**Figure 2:**
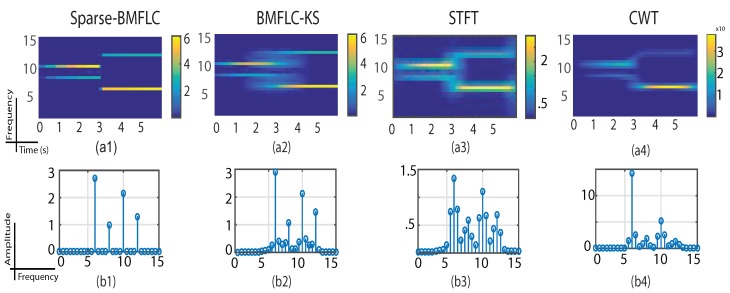
Time-frequency mapping for $S_{1}\left(t\right)$. (**a1**–**a4**) are the time-frequency mapping obtained from sparse-BMFLC, BMFLC-KS, STFT and CWT, respectively. The corresponding spectra shown in (**b1**–**b4**) are obtained by averaging the time-frequency mapping over time.

The time-frequency decomposition of $S_{1}\left(t\right)$ for all methods is shown in Figure 2. $S_{1}\left(t\right)$ has a frequency transition that occurs at 3 s. As BMFLC-KS relies on the estimation obtained from the Kalman filter, it also inherits the disadvantages of the adaptive filter with regards to the delay in settling to its steady-state. As shown in Figure 2(a2), the BMFLC-KS took approximately 1 s to settle to the new frequency components. As expected, the estimated amplitude of the time-frequency mapping was blurred in both directions at the frequency transition.

Sparse-BMFLC, STFT and CWT can detect the sudden frequency changes without any lag. However, the amplitude estimation from sparse-BMFLC is superior as compared to STFT and CWT. From Figure 2(a1), we can observe that sparse-BMFLC tracks the modulated amplitude accurately as indicated by the gradual color intensity change during the initial stage. The flat average spectrum that is shown in Figure 2(b1) further demonstrates the superior performance of sparse-BMFLC compared to other methods.

Although STFT and CWT can identify the dominant frequency components in the signal as shown in Figure 2(a3,a4), the leakage effect in amplitude estimation is also evident as shown in Figure 2(b3,b4). STFT shows several frequency components between 5 Hz to 12.5 Hz, although there are only four frequency components present in the signal. The flat average spectrum shown in Figure 2(b4) further shows that CWT underestimated the two frequency components. The performance of CWT highly depends on the selected mother wavelet and the parameters selected for the mother wavelet. The insufficiency in tracking the dynamic changes of CWT can partially be attributed to the fixed parameters in the CWT implementation. In contrast, the BMFLC-based model only requires the knowledge of signal frequency band to be known a priori.

**Figure 3:**
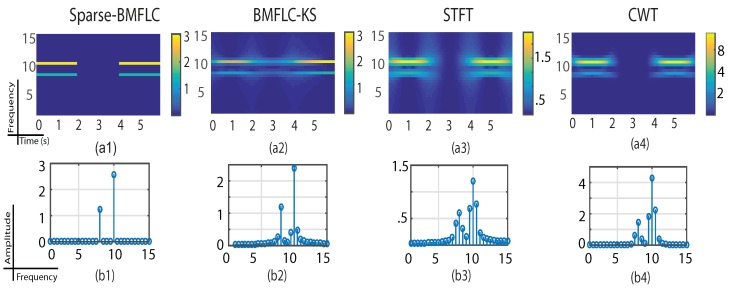
Time-frequency mapping for $S_{2}\left(t\right)$. (**a1**–**a4**) are the time-frequency mapping plots obtained for sparse-BMFLC, BMFLC-KS, STFT and CWT, respectively. The corresponding spectra shown in (**b1**–**b4**) are obtained by averaging the time-frequency mapping over time.

The time-frequency mapping of $S_{2}\left(t\right)$ obtained for all algorithms are shown in Figure 3. The middle section of $S_{2}\left(t\right)$ is set to zero to test the temporal resolution of the algorithms. The zeros in the signal also mimic the scenario where partial data is missing. Among all, the BMFLC-KS does not fare well as shown in Figure 3(a2), whereas sparse-BMFLC provides the best performance Figure 3(a2). One can notice that BMFLC-KS fails to correctly estimate the signal amplitude when the amplitude is zero. The spectral leakage effect of STFT is clearly visible in Figure 3(a3) as the amplitude spreads to the whole time-frequency plane when the signal amplitude suddenly vanishes. It is interesting to note that the results obtained from CWT has good temporal resolution as shown in Figure 3(a4). Compared to the results of CWT obtained for $S_{1}\left(t\right)$, we can note that the parameter selection for Morlet mother wavelet emphasizes more the temporal resolution rather than the spectral resolution. From the average spectra shown in Figure 3(b1–b4), compared to the rest, sparse-BMFLC has the best performance in suppressing the leakage effect. The sharp peaks found in Figure 3(b1) are well in line with the frequency components that are present in $S_{2}\left(t\right)$.

**Figure 4:**
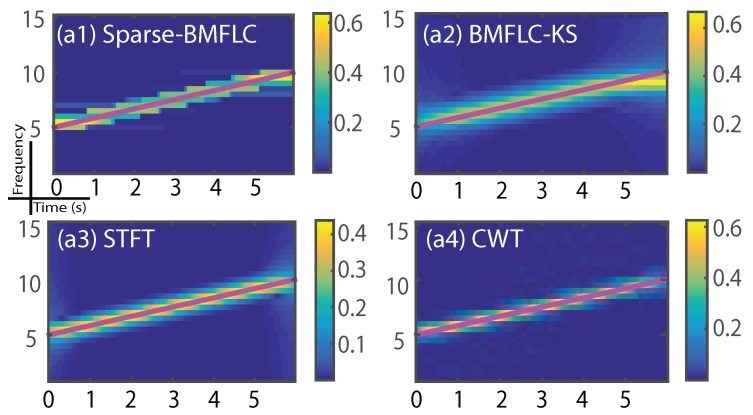
Time-frequency mapping for $S_{3}\left(t\right)$. (**a1**–**a4**) are the time-frequency mapping obtained from sparse-BMFLC, BMFLC-KS, STFT and CWT, respectively.

To demonstrate the robustness of the proposed method, a chirp signal that contains frequency components that are not modelled by BMFLC is employed to analyze the case of frequency mismatch. The magenta line in Figure 4 indicates the true frequency sweeping pattern. It is clear from the results that all methods show amplitude estimates to the frequency components around the magenta line as shown in Figure 4(a1–a4). As the sparse-BMFLC employed the $\ell_{1}$ norm to ensure that less number of coefficients are non-zero, and the changes in its amplitude estimates are expected to be in a block-wise manner as shown in Figure 4(a1). The BMFLC-KS, STFT and CWT show a blurred area around the true frequency as shown in Figure 4(a2–a4), respectively. CWT provides better performance as shown by the smallest deviation between the estimated frequency and the true frequency.

Furthermore, to quantify the performance of time-frequency decomposition of various algorithms, an energy ratio metric is employed. The energy ratio measures the amplitude discrepancy between the estimated time-frequency mapping and the amplitude of the true frequency components that are present in the signal. Recalling that the time-frequency mapping of a signal is defined as P(t,f), the energy ratio metric can be defined as

(16) $$\begin{array}{c} R=\frac{1}{N}\sum_{t=1}^{t=N}\left(\frac{\sum_{f_{j}\in f^{*}}P\left(t,f_{i}\right)}{\sum_{i=1}^{i=M}P\left(t,f_{i}\right)}\right), \end{array}$$

where $f^{*}$ is the set of true frequency components that are present in the signal, *M* and *N* are the number of total frequency components in the model and total number of samples of the signal, respectively. If the detected frequency components match the true frequency components in the signal, the value of the energy ratio metric equals 1, while a total mismatch produces a value of 0. Note that this metric can only be applied in a scenario where the true frequency components are known. Hence, we have excluded $S_{3}\left(t\right)$ in this analysis. The results obtained for energy ratio metric are tabulated in Table 2. The energy ratio again confirms the superiority of sparse-BMFLC in time-frequency decomposition. The BMFLC-KS performs better than STFT and CWT. The CWT outperforms STFT on all signals. The leakage effect of STFT is the reason for its sub-par performance.

**Figure 5:**
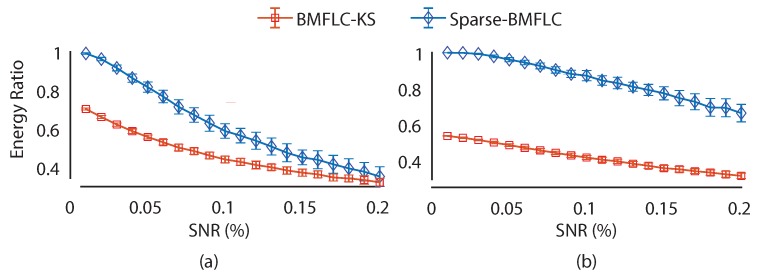
Performance of Sparse-BMFLC and BMFLC-KS under different signal-to-noise ratio levels. (**a**) $S_{1}\left(t\right)$; (**b**) $S_{2}\left(t\right)$.

To demonstrate the stability of Sparse-BMFLC under noisy conditions, the synthetic signals $S_{1}\left(t\right)$ and $S_{2}\left(t\right)$ with additive Gaussian noise were employed. The amount of Gaussian noise was determined by the signal-to-noise ratio (SNR), which is defined as $SNR=\frac{\delta_{Signal}}{\delta_{Noise}}$, where δ denotes the variance of a signal. We varied SNR from 0.01 to 0.2 with a step size of 0.01. At each SNR level, Gaussian noise with variance equals $\delta_{Noise}=\delta_{Signal}*SNR$ was added to the original signal. Time-frequency decomposition of the noise contaminated signal was obtained by using Sparse-BMFLC and BMFLC-KS, respectively. With 100 realizations of Gaussian noise at each SNR level, the mean and standard deviation of energy ratio for both BMFLC based algorithms were estimated, and it was shown in Figure 5.

Overall, the mean energy ratio of Sparse-BMFLC and BMFLC-KS decreases with increase in SNR level for both synthetic signals. As shown in Figure 5a,b, the proposed Sparse-BMFLC outperforms the BMFLC-KS in mean energy ratio over all SNR levels for both signals. The results indicate that the sparsity constraint employed in Sparse-BMFLC could help in providing an accurate time-frequency decomposition as compared to BMFLC-KS, and the proposed Sparse-BMFLC also has good robustness to noise contamination. However, as each realization of Gaussian noise distorts the frequency components of the original signal differently, it further causes the optimal solution of Sparse-BMFLC to vary in each run, which results in the larger standard deviation that is observed Figure 5a,b. As in the case of BMFLC-KS, the Gaussian noise can be canceled by the employed Kalman smoother; therefore, the optimal solutions of BMFLC-KS are similar at each run. Nonetheless, the performance of time-frequency decomposition is still superior when Sparse-BMFLC is employed. The difference in standard deviation only reflects the effects of optimization algorithms for solving BMFLC-based models.

### 3.5. Time-Frequency Decomposition of Respiratory Motion Signal

As a case study, we have tested the proposed Sparse-BMFLC and BMFLC-KS for obtaining time-frequency decomposition of the respiratory motion signal. Respiratory rate (RR) estimated from respiratory motion signal varies under different physiological conditions such as sleep [35], exercise [36] and anesthesia [37]. An accurate track of RR changes in time-frequency domain is required and can provide insight into functioning of the automatic nervous system [38,39].

**Figure 6:**
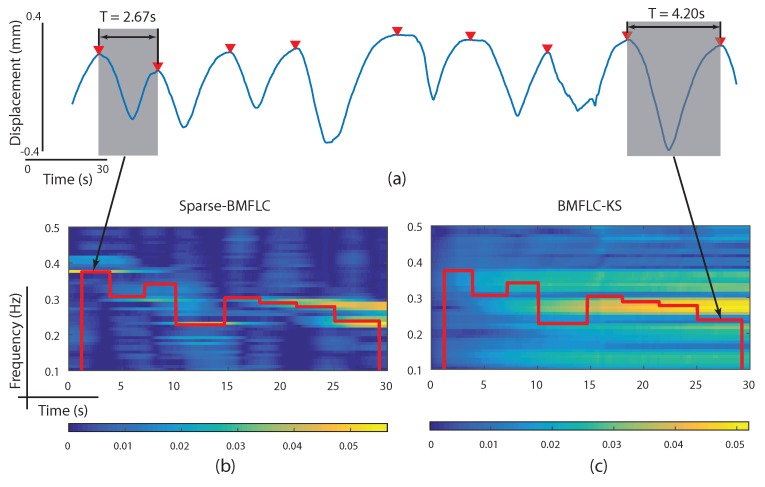
Time-frequency decomposition of respiratory motion signal. (**a**) raw respiratory motion signal; (**b**) time-frequency mapping obtained from Sparse-BMFLC; (**c**) time-frequency mapping obtained from BMFLC-KS.

A respiratory motion signal of 30 s duration during normal breathing conditions was selected for testing the proposed Sparse-BMFLC and BMFLC-KS. The data was collected with optical sensors placed on the subject’s chest and recorded by infrared cameras. For a detailed experiment procedure, please refer to [40]. The raw respiratory motion signal is shown in Figure 6a.

To quantify the performance of different algorithms, the RR and its corresponding frequency were estimated. To do so, we first identified the peak position of a given respiratory circle marked with a red arrow as shown in Figure 6a. The RR was defined as the time duration between two consecutive peaks, and its corresponding frequency was calculated by taking the reciprocal of RR. The obtained frequency estimation for each respiratory circle was then superimposed on the time-frequency maps obtained from both Sparse-BMFLC and BMFLC-KS as shown Figure 6b,c. It is evident that the RR and its corresponding frequency estimation varies slightly for each respiratory circle. Comparing the time-frequency map obtained from Sparse-BMFLC and BMFLC-KS, it is clear that Sparse-BMFLC can accurately track the changes in RR, whereas the time-frequency map obtained with BMFLC-KS was blur. This result further demonstrates the superiority of Sparse-BMFLC in obtaining time-frequency decomposition as compared to BMFLC-KS.

## 4. Discussion

Although, by construction, the performance of BMFLC based methods depends on the prior knowledge of the frequency characteristics of the signal, and the results obtained with the chirp signal show that the proposed model was able to tolerate the discrepancy to a certain extent and provide a reasonable time-frequency mapping. However, as our results suggest, if the frequency mismatch is suspected in the signal, BMFLC-KS should be employed instead of sparse-BMFLC.

The proposed sparse-BMFLC relies on the optimization algorithm to estimate the amplitude and the frequency in the model. However, the sparse-BMFLC can provide better temporal and spectral resolution than the other methods in comparison, the proposed method is computationally more expensive as compared to other existing methods. In comparison, STFT and CWT do not require much computational power for estimation of a large number of frequency components. Hence, the proposed method is more suitable for band-limited signals when more detailed and accurate time-frequency decomposition is required for analysis.

## 5. Conclusions

In this paper, a truncated Fourier linear combiner model was re-formulated in the form of sparse linear regression. Results show that the coefficients of sparse-BMFLC estimated with the ADMM algorithm can be used to reconstruct the original signal with a high degree of accuracy. The frequency tracking study also showed that the sparse-BMFLC can successfully track the signal with amplitude modulation. Furthermore, the proposed method outperforms BMFLC-KS, STFT and CWT in temporal and spectral resolutions of the time-frequency mapping. With the energy ratio metric, the overall time-frequency decomposition performance for all methods was quantified. The results show that proposed sparse-BMFLC has a high level of accuracy in both time and frequency domains. Furthermore, the energy ratio also clearly demonstrates the absence of leakage effect in the proposed method, which can be found in the traditional methods such as STFT and CWT. With the noise contaminated signals, we showed that the proposed Sparse-BMFLC has good robustness to noise over a wide range of SNRs. The benefits of employing sparsity constraints were also highlighted by the superior performance of the proposed method for all SNR levels. With a case study on respiratory motion signal, we have illustrated that the Sparse-BMFLC can identify the subtle changes in the spectrum. It further highlights the suitability of the approach for biological/biomedical applications.

## Figures and Tables

**Table 1:** Reconstruction error for BMFLC based methods.

Methods	$S_{1}\left(t\right)$	$S_{2}\left(t\right)$	$S_{3}\left(t\right)$
BMFLC-KS	2.12%	3.01%	3.00%
Sparse-BMFLC	5.12%	3.95%	9.59%

**Table 2:** Performance analysis of all methods: Energy ratio perspective.

Synthetic Signal	BMFLC-KS	Sparse-BMFLC	CWT	STFT
$S_{1}\left(t\right)$	0.7328	0.9972	0.5961	0.4151
$S_{2}\left(t\right)$	0.5596	0.9981	0.4819	0.3492
